# Immunotherapy in prostate cancer: new horizon of hurdles and hopes

**DOI:** 10.1007/s00345-020-03497-1

**Published:** 2020-10-26

**Authors:** Igor Tsaur, Maximilian P. Brandt, Eva Juengel, Cécile Manceau, Guillaume Ploussard

**Affiliations:** 1 Department of Urology and Pediatric Urology, University Medical Center, Johannes Gutenberg University, Langenbeckstr. 1, 55131 Mainz, Germany; 2 Department of Urology, CHU-Institut Universitaire du Cancer Toulouse—Oncopole, Toulouse, France; 3 Department of Urology, La Croix du Sud Hospital, Toulouse, France

**Keywords:** Prostate cancer, Immunotherapy, Immune checkpoints, Vaccine, PD-1, PD-L1

## Abstract

**Purpose:**

Prostate cancer (PCa) is the most common malignancy in men and the cause for the second most common cancer-related death in the western world. Despite ongoing development of novel approaches such as second generation androgen receptor targeted therapies, metastatic disease is still fatal. In PCa, immunotherapy (IT) has not reached a therapeutic breakthrough as compared to several other solid tumors yet. We aimed at highlighting the underlying cellular mechanisms crucial for IT in PCa and giving an update of the most essential past and ongoing clinical trials in the field.

**Methods:**

We searched for relevant publications on molecular and cellular mechanisms involved in the PCa tumor microenvironment and response to IT as well as completed and ongoing IT studies and screened appropriate abstracts of international congresses.

**Results:**

Tumor progression and patient outcomes depend on complex cellular and molecular interactions of the tumor with the host immune system, driven rather dormant in case of PCa. Sipuleucel-T and pembrolizumab are the only registered immune-oncology drugs to treat this malignancy. A plethora of studies assess combination of immunotherapy with other agents or treatment modalities like radiation therapy which might increase its antineoplastic activity. No robust and clinically relevant prognostic or predictive biomarkers have been established yet.

**Conclusion:**

Despite immunosuppressive functional status of PCa microenvironment, current evidence, based on cellular and molecular conditions, encourages further research in this field.

## Introduction

Despite promising results of immunotherapy (IT) in genitourinary malignancies such as urothelial and kidney cancer, IT has not turned out to be a meaningful player in the treatment armamentarium of advanced prostate cancer (PCa) yet. The only registered agent in the field, sipuleucel-T, an immunostimulant based on dendritic cells, has shown a benefit in overall survival (OS) of almost 4 months compared to placebo in metastasized castration-resistant PCa (mCRPC) [1]. However, a serious drawback was that a viral vector-based IT approach reported in the PROSTVAC-trial could not show any positive effect on OS in the most recent update [2]. In addition, development of novel treatment strategies such as androgen receptor targeted therapies (ART) has shifted the clinical focus somewhat away from IT in advanced PCa.

Bearing in mind the robust advances made with the development of programmed cell death ligand-1 (PD-L1) and programmed cell death-1 receptor (PD-1) inhibitors in a number of solid malignancies, immune oncology remains an essential part of the current research activities in PCa, despite the fact that PCa is considered a non-immunoreactive and a “cold” tumor with an immunosuppressive tumor microenvironment (TME) and low infiltration burden of T cells. For instance, pembrolizumab is presently under investigation in combination with several other standard of care regimens such as secondary ARTs (ClinicalTrials.gov: NCT02787005) or poly(ADP-ribose) polymerase (PARP) inhibitors (ClinicalTrials.gov: NCT03834519).

Within the complexity of the immune system and involvement of a multitude of immune cells, key enzymes and receptors, the immunotherapeutic approach remains a highly appealing strategy to optimize treatment for patients with PCa. In this review, we highlight the current underlying mechanisms of immunotherapy in cancer, with a focus on PCa. Furthermore, we provide an overview of the relevant clinical trials that have the potential to reshape the landscape of PCa treatment in the near future.

## Materials and methods

Between June 26 and July 11, 2020, we searched Medline, Embase and other databases as well as the Google web search engine for peer-reviewed articles and published abstracts from international congresses in English using the terms “prostate cancer” and “immune therapy”, “immunotherapy”, “immune-oncology drug”, “vaccine” as well as “checkpoint inhibitor”. Furthermore, we searched ClinicalTrials.gov for clinical trials evaluating immunotherapy in PCa that have been recently completed, are ongoing or are actively recruiting participants.

## Molecular aspects of the immune contexture in prostate cancer

### Tumor microenvironment and infiltrating immune cells

Cancer formation and progression strongly depend on the TME in which it develops [3]. Besides tumor cells, solid malignancies are composed of a number of other cells including fibroblasts, endothelial cells, innate and adaptive immune cells, extracellular matrix as well as extracellular soluble molecules like cytokines, chemokines, growth factors and metabolic products [4]. Importantly, PCa is known to be a "cold" tumor with a low T-cell infiltration. Thereby, effective immune response counteracting tumor progression presupposes activation of cancer-combatting host immune cells, their enrichment at the tumor sites and overcoming the dormant impact of tumor-associated immunosuppressive cells on the TME, mediated by secreted and cellular factors [5]. In an attempt to establish a tumor-agnostic, prognostic and predictive biomarker based on the immune contexture, “Immunoscore” has been developed in the area of colorectal cancer [6–8]. It relies on the quantification of lymphocyte populations, in particular CD3^+^ and CD8^+^ T cells, counted at the tumor center and at the invasive margin. Thereby, increasing score correlates with a longer patient survival [3] and was also supposed to predict response to immune checkpoints inhibitors targeting PD-1/PD-L1 or CTLA4 [7, 9]. Another approach to predict response to immunotherapy is the “tumor inflammation signature” (TIS). This 18-gene signature measures the level of T-cell inflammation as an immune-phenotyping tool across different histologically defined tumor types [10]. Analysis of 9,083 samples of 32 cancers, including PCa, demonstrated that tumors with a known clinical sensitivity to PD-1 blockade had a higher TIS average [10]. On the whole, utilization of these tools allows to classify solid malignancies into T-cell inflamed/“hot” and non-T-cell inflamed/“cold tumors” [5, 10].

Immune contexture determined by the density, composition, functional state and organization of the tumor infiltrating immune cells can yield information relevant for prognosis and treatment response [11]. For example, a positive association of the level of stromal tumor infiltrating lymphocytes (TILs) with adjuvant chemotherapeutic response in ovarian high-grade serous carcinoma has been observed [12]. Moreover, Shibutani and collaborators presented evidence for a significantly higher chemotherapeutic response rate and better progression-free survival in patients with stage IV colorectal cancer revealing a high number of TILs [13]. Indeed, the presence of cytotoxic and helper T cells within the tumor center or invasive margin has been linked to favorable outcomes in a plethora of malignancies [5, 11]. Idos et al. reported favorable outcomes in colon cancer when high levels of TILs, presence of CD3^+^, CD8^+^ and FOXP3 + cells at the tumor center and CD3^+^ at the invasive margin of the tumor, were observed [14]. In line with this, an association of a high tumor CD8^+^ T-cell density with a longer overall survival in non-small cell lung cancer has been demonstrated [15]. However, opposing results have also been reported. Kim and coauthors have recently demonstrated that a high CD8^+^ expression predicted independently for a shorter disease-free survival of breast cancer patients [16]. Triozzi et al. postulated a crucial role of CD8^+^ regulatory T cells in fostering uveal melanoma disease progression, while TIL infiltration correlated with a worse prognosis in this disease [17]. Thus, impact of CD8^+^ TILs might be entity specific or depend on additional factors.

### Tumor microenvironment and prostate cancer

Observations regarding prognostic value of CD8^+^ TIL infiltration in PCa are contradictory. Ness and coauthors demonstrated that a high density of CD8^+^ TILs in the primary PCa specimens is an independent negative prognostic factor for biochemical failure-free survival [18]. In concert with this, a high density of CD8^+^ TILs and PD-L1 expression by tumor cells has been associated with a higher risk of clinical progression in men with node-positive PCa [19]. On the contrary, Yang et al. recently demonstrated that a high number of CD8^+^ TILs at prostatectomy is independently associated with improved survival in this majority of a high-risk PCa population [20]. In line, Vicier and collaborators reported that a high PD-L1 and low CD8^+^ TIL density are markers for poor prognosis and biochemical and metastatic relapse in PCa [21]. Thus, aside CD8^+^, additional parameters seem to be important for prognosis. Indeed, the ability of CD8^+^ effector T cells to promote tumor regression is largely dependent on their cytokine secretion profile and their ability to self-renew [22]. Emerging evidence demonstrates that the TME can provoke emergence of dysfunctional CD8^+^ T cells with a limited cytotoxic function [22]. Furthermore, senescent, regulatory, and dysfunctional stem-cell like memory CD8^+^ T-cell phenotypes, which do not exert antitumor activity, might coexist. Thus, a deeper profiling of the functional status and subsets of infiltrating immune cells, beyond a general surface labelling, and their spatial distribution, is required to utilize TILs as a predictive or prognostic marker.

The same holds true for different types of tumor-associated macrophages. PCa cells and cancer-associated fibroblasts stimulate monocyte recruitment toward tumor cells and their trans-differentiation into anti-inflammatory and tumor-promoting M2 macrophage phenotype [23]. Comito and collaborators yielded evidence for a more favorable biochemical recurrence-free survival of PCa patients with M1 macrophage (pro-inflammatory and tumor-suppressive) prevalence in prostatectomy specimens, compared to those with M2 macrophage prevalence [23]. TGF-beta, typically secreted by M2 macrophages, promotes various tumorigenic processes like recruitment of mesenchymal stem cells, their activation into cancer-associated fibroblasts, or PI3K-AKT signaling, fostering migration of PCa cells [24, 25]. As TGF-beta is important for immune exclusion, it might represent one essential aspect of a reduced infiltration of TILs and immunosuppressive TME in PCa [26].

### Tumor mutational burden and PD1/PD-L1 signaling

PCa is characterized by a low tumor mutational burden (TMB), thus revealing a poor collection of neoepitopes crucial for immune cell attraction to the tumor sites, epitope–MHC interactions and activation of TILs by antigen-presenting cells [5, 27]. PCa has distinctly fewer mutations (0.7 per Mb) than breast (1.2 per Mb), bladder (7.1 per Mb) and colorectal cancer (3.1 per Mb), or melanoma (12.1 per Mb) [28]. Even in castration-sensitive or -resistant disease, TMB is only as high as 2.08 and 4.02 per Mb, respectively [29]. Due to a low TMB and T-cell-mediated inflammation, the probability that PCa responses to anti-PD1/PD-L1 treatment is weak [30].

It has been speculated that PCa in general is associated with a low expression of PD-L1 due to few effector T cells secreting proinflammatory cytokines [31]. Analysis of primary PCa specimens by Xian and coworkers revealed only 17.9% PD-L1 positivity [32]. In males with advanced tumor stage, lymph node metastasis, and high Gleason score more PD-L1^+^ tumors were found. Moreover, Haffner et al. demonstrated that PD-L1 positivity counts 7.7% in primary PCa, while it increased to 31.6% in mCRPC [33]. Bishop and coauthors showed that patients progressing on enzalutamide had a higher number of PD-L1/2^+^ dendritic cells in blood compared to those naïve or responding to treatment, and a high frequency of PD-1 + T cells [34]. In contrast to this, tumor specimens from men with intermediate- to high-risk PCa pretreated with abiraterone acetate prior to radical prostatectomy yielded less CD8^+^ T cells and a trend for decreased PD-L1 positivity (7% vs. 21%; *p* = 0.062), compared to untreated PCas [35]. Thus, there are considerable differences in the expression of PD-1/PD-L1 in PCa depending on tumor stage, previous treatment, and methodological issues. Further research is warranted to clarify and generalize prognostic and predictive value of these immune checkpoints.

### HLA alteration

Attenuating HLA class I proteins, which are commonly abundant on nucleated cells and present intracellular peptides to T lymphocytes, is an established escape mechanism of tumor cells from cytotoxic T cells in different cancers, and associated with unfavorable clinical course and resistance to immunotherapy [36]. A complete loss and, in case of individual allelic expression, a minimal estimated downregulation of HLA class I in 34 and 85% of primary PCas and in 80 and 100% of lymph node metastases, respectively, has been shown [37]. Moreover, downregulation of several components of HLA class I antigen processing machinery and association with a higher Gleason score and an early disease recurrence has been reported in PCa [38]. Interestingly, treating PCa cells with IFN-γ, crucial for efficient antitumor immune response and normally secreted by cytotoxic T lymphocytes, resulted in upregulation of HLA class I [39, 40]. Similarly, reversion of defects in HLA class I expression and survival improvement by IFN-γ treatment in a mouse model of PCa was demonstrated [41]. Whether therapeutically induced augmentation of the expression of HLA class 1 proteins in humans may shift the functionally immunosuppressive TME with a low TIL density towards immunoactive setting remains questionable [5]. Taken together, tumor progression and patient outcomes depend on complex cellular and molecular interactions of the tumor with the host immune system [42].

## Clinical utilization of immunotherapy in prostate cancer and future directions

Currently, IT is used for patients with specific mutations but also for general PCa population, alone or in combination. A selection of phase 2 and 3 studies is shown in Table 1.

**Table 1:** Main phase 2 and 3 trials of vaccination and oral immunotherapy drugs in prostate cancer management

Clinical trial	Phase	Treatment	Patients	Population	Endpoints	Follow-up (months)	OS (months)	Factors favoring drug (subgroup analysis)	Conclusion	Safety profile
Vaccines										
Sipuleucel-T [1]	3	Sipuleucel-T vs placebo (2:1)	512	mCRPC with expected survival > 6 months	I. OS II. Objective disease progression	34.1	25.8 vs 21.7	N/A	I. 25.8 vs 21.7 (HR = 0.78, 95% CI 0.61–0.98 *p* = 0.003) II. median time to objective disease progression 14.6 vs 14.4 (HR = 0.95; 95% CI, 0.77–1.17; *p* = 0.63)	Grade 3–5 AE: 31.7% vs 35.1%
Sipuleucel-T (D9901 and D9902A) [2]	3	Sipuleucel-T vs placebo (2:1)	225	Asymptomatic mCRPC	I. time to disease progression II. OS	> 36	23.2 vs 18.9	N/A	I. 11.1 vs 9.7 (HR = 1.26 95% CI 0.95–1.68; *p* = 0.111) II. 23.2 vs 18.9 (HR = 1.50 95% CI 1.10–2.05; *p* = 0.011)	Grade 3–5 AE: ≤ 5% in 2 groups without difference
Sipuleucel-T (APC8015) [3]	3	Sipuleucel-T vs placebo (2:1)	127	Asymptomatic mCRPC	I. time to disease progression II. OS	> 36	24.9 vs 21.4	N/A	1. 11.7 vs 10.0 (HR = 1.45 95% CI 0.99–2.11; *p* = 0.052) II. 24.9 vs 21.4 (HR 1.70 95% CI 1.13–2.56; *p* = 0.01)	Any AE: 95.1 vs 93.3 (*p* ≤ 0.05) Grade 3–4 AE: 24.4% vs 24.4%
PROSTVAC [4]	2	PROSTVAC vs placebo (2:1)	125	Minimally symptomatic mCRPC	I. PFS II. OS	41.3	25.1 vs 16.6	N/A	I. median PFS 3.8 vs 3.7 (HR = 0.884, 95% CI 0.568–1.375, *p* = 0.60) II. OS 25.1 vs 16.6 (HR 0.56 95% CI 0.37–0.85; *p* = 0.0061)	Mild injection site reactions
PROSTVAC [5]	3	PROSTVAC + placebo vs PROSTVAC + GMCSF vs placebo + placebo (1:1:1)	1297	Minimally or asymptomatic mCRPC	I. OS II. PFS at 6 months	N/A	34.4 vs 33.2 vs 34.3	N/A	I. ns placebo vs PROSTVAC placebo 34.3 vs 34.4 (HR = 1.01 95% CI 0.84–1.20; *p* = 0.47) Placebo vs PROSTVAC + GMCSF 34.3 vs 33.2(HR 1.02 95%CI 0.86–1.22; *p* = 0.59) II. ns	Grade 3–4 AE: 3.3% vs 4.7% vs 2.6%
Checkpoint inhibitor										
Ipilimumab (NCT02113657) [6]	2	Ipilimumab 3 mg/kg	30	mCRPC	PSA PFS rPFS OS	45.5	24.3	Favorable cohort: higher density of cytotoxic and memory T cells in the tumor and increased expression of interferon-γ signaling	PSA PFS: 1.7 months rPFS: 3.0 months OS: 24.3 months Favorable cohort (*n* = 9) rPFS > 6 months and OS > 12 months	Grade 3–4 AE: 28% No death
Ipilimumab (CA184-095) [7]	3	Ipilimumab 10 mg/kg vs placebo (1:1)	602	mCRPC without visceral metastasis and chemotherapy naive	I. OS II. PFS	NA	27.8 vs 29.7	N/A	I. 27.8 vs 29.7 months (HR = 1.11; 95% CI, 0.88–1.39) *p* = 0.3667 II. 5.6 vs 3.8 months (HR = 0.67; 95% CI, 0.55–0.81) p	grade 3–4 AE: 27% vs 2% Deaths because of AE: 2% vs 0%
Pembrolizumab (KEYNOTE-199) [10]	2	Pembrolizumab 200 mg	260	mCRPC Cohort 1: PD-L1 positive (*n* = 133) Cohort 2: PD-L1 negative (*n* = 66) Cohort 3: bone predominant disease *n* = 59)	I. ORR II. DCR OS PSAr	16.8	Cohort 1: 9.5 Cohort 2: 7.9 Cohort 3: 14.1	N/A	I. Cohort 1: 5% Cohort 2: 3% II. DCR Cohort 1: 10% Cohort 2: 9% Cohort 3: 22% OS Cohort 1: 9.5 months Cohort 2: 7.9 months Cohorte 3: 14.1 months PSAr: Cohorte 1: 6% Cohorte 2: 8% Cohorte 3: 22%	60% AE Grade 3–5 AE: 15%
Combination										
Nivolumab + Ipilimumab CheckMate 650 [12]	2	Nivolumab 1 mg/kg + ipilimumab 3 mg/kg	78	mCRPC Cohort 1: symptomatic or minimally symptomatic patients, progression after second generation hormone therapy and no chemotherapy Cohort 2: Progression after taxane-based therapy	ORR rPFS	> 6		PD-L1 ≥ 1%, DNA damage repair Homologous recombination deficiency tumor mutational burden above median	ORR 26% in cohort 1, 10% in cohort 2	Grade 3–4 AE: 39% cohort 1 51% cohort 2 Death: one in each cohort
Nivolumab + Ipilimumab [13]	2	Nivolumab 1 mg/kg + ipilimumab 3 mg/kg	15	mCRPC ARV7 +	I. PSAr II. ORR Durable PFS (> 24 weeks) PSA-PFS rPFS OS	8.4	9.5	DNA repair deficient tumors	I. PSAr: 7% II. ORR 25% durable PFS rate:20% PSA-PFS 3.0 months rPFS 3.9 OS 9.5	Grade 3–5: 46% No death
GVAX + ipilimumab [14]	1/2	GVAX (13 biweekly) + ipilimumab (escalating dose 0.3, 1, 3, and 5 mg/kg)	28	mCRPC asymptomatic chemo naive	PSAr		29	Treatment induces: > 25% increases lymphocyte counts > 30% increases non-naive (memory) CD4 + T cells CD4 + and CD8 + T-cell activation Pre-treatment: high frequencies of CD4 + CTLA-4 + , CD4 + PD-1 + , non-naive CD8 + low frequencies of CD4 + or regulatory T cells	PSA partial decline: 32.1% PSA stable disease: 42.8%	32% AE
Atezolizumab + enzalutamide (IMbassador250) [15]	3	Atezolizumab1200mg + enzalutamide. 160 mg vs enzalutamide 160 mg alone (1:1)	759	mCRPC who had progressed after chemotherapy	I. OS II. PSAr rPFS ORR		15.2 vs 16.6	N/A	I. 15.2 vs 16.6 months (HR = 1.12, 95% CI 0.91–1.37) *p* = 0.28 II. ongoing	Grade 3–4 AE: 28.3% vs 9.6% Death: 1.9% vs 0.3%
Pembrolizumab + enzalutamide (KEYNOTE-199) [16]	2	Pembrolizumab 200 mg + enzalutamide 160 mg	126	mCRPC chemotherapy naïve who had progressed with enzalutamide Cohort 4: RECIS-mesurable disease Cohort 5: bone-predominant disease	I. ORR II. DCR PSA r rPFS OS	13.7	Cohort 4: N/A Cohort 5: 19		I. Cohort 4:12% Cohort 5: NA II. DCR Cohort 4:51% Cohort 5: 51% PSAr Cohort 4:17% Cohort 5: 9% rPFS Cohort 4: 4 months Cohort 5: 4 months	Grade 3–5 AE: Cohort 4: 26% Cohort 5: 24% Deaths: 2 patients in cohort 4
Durvalumab + olaparib [17]	2	Durvalumab 1500 mg + olaparib 300 mg/12 h	17	mCRPC who had progressed after 2^nd erscript> generation hormonotherapy^	PSAr rPFS			Alteration in DDR: 12 months PFS probability 83.3% with alteration DDR mutation vs 36.4% without	PSAr: 53% rPFS: 16.1 months	Most common grade 3–4 AE: anemia 24% lymphopenia 12% infection 12%, nausea 12%
Ipilimumab + radiotherapy [18]	1/2	Ipilimumab 10 mg/kg ± bone directed therapy (n = 34)	50	mCRPC	Safety RECIST PSA decline > 50%	15.7	17.4	NA	RECIST: Stable 21.4% Partial 7.1% Complete 3.6% PSA decline > 50%: 16%	Grade 3–4: 32%
Ipilimumab + radiotherapy (CA184-043) [19]	3	Bone directed therapy + Ipilimumab 10 mg/kg or placebo (1:1)	799	mCRPC with bone metastasis who had progressed after docetaxel	I. OS II. PFS	9.9 and 9.3	11.2 vs 10.0	Race white ECOG score 0 ALP < 1.5 N Gleason score > 7 Normal LDH level No visceral metastases Hemoglobin > 110 g/L Not North America region Low pain score	I. 11.2 vs 10.0 months (HR = 0.85, 0.72–1.00) *p* = 0·053 II. 4.0 vs 3.1 months [HR 0.70, 95% CI 0.61–0.82] *p* < 0.001	Grade 3–4 AE: 59% vs 41% Deaths AE: 17% vs 11%
Pembrolizumab + androgen deprivation + prostate cryotherapy [20]	Pilot trial	Pembrolizumab 200 mg	13	Oligometastatic hormone sensitive PCa	I. PSA < 0.6 ng/mL at 1 year II. PSA PFS Systemic therapy FS CRPC FS	31.3			I. 42% II. median PSA PFS 14 months Median systematic therapy FS survival: 17 months CRPC FS: not reached	No grade 3–4 AE No death

*AE* adverse effect; *ALP* alkaline phosphatase; *CI* confidence interval; *CRPC FS* castration-resistant prostate cancer-free survival; *DDR* DNA damage repair; *DCR* disease control rate; *ECOG* Eastern Cooperative Oncology Group; *HR* hazard ratio; *LDH* lactate deshydrogenase;* mCRPC* metastatic castration resistant prostate cancer; *ORR* objective response rate; *OS* overall survival; *PSAr* PSA response*; PSA PFS* PSA progression-free survival; *rPFS* radiologic progression free survival

### Vaccines

Active cellular ITs, named therapeutic cancer vaccines, have been tested as PCa therapy. Sipuleucel-T is a personalized therapy, made from patient’s peripheral blood mononuclear cells incubated with a fusion protein consisting of a common prostate cancer antigen (prostatic acid phosphatase) linked to an adjuvant (granulocyte–macrophage colony-stimulating factor). Infused into the patient, it induces CD4^+^ and CD8^+^ immune cells against the tumor antigen. The main phase 3 trial randomly assigned 512 patients with mCRPC and an expected survival of at least 6 months to receive sipuleucel-T or placebo [1]. With 34.1 months median follow-up, OS was 25.8 months in the sipuleucel-T group vs 21.7 in placebo group. However, there was no difference regarding the time to objective disease progression (14.6 in sipuleucel-T vs 14.4 months in placebo groups, respectively). These results were consistent with two previous phase 3 studies [43, 44]. To date, sipuleucel-T remains the only approved vaccine therapy for PCa. Importantly, this medicine is withdrawn from the use in Europe. All-in-all, its complex administration, high price, supply problems due to a limited manufacturing capacity and uncertainty about the reimbursement status hampered its prescription resulting in bankruptcy of its owner Dendreon [45].

PROSTVAC utilizes recombinant poxviruses that express PSA with immune-enhancing costimulatory molecule to stimulate immune response. A phase 2 trial showed a median survival improvement of 8.2 months (*p* = 0.0061) although this was not the primary endpoint [46]. Subsequently, a phase 3 trial [2] did not support the initially positive signal of the phase 2 study with no effect on OS and progression free survival (PFS) in mCRPC patients.

GVAX consists of two metastatic prostate cancer cell lines transfected with a human *GM-CSF* gene. GVAX injection breaks immune tolerance to antigens expressed by prostate cancer and induces antitumor immune responses. Two phase 3 trials have been initiated (VITAL1 and 2) to compare GVAX to docetaxel plus prednisone in asymptomatic and symptomatic metastatic prostate cancer patients but were both stopped for futility and an increase in mortality.

### Checkpoint inhibitors

Ipilumumab is a CTLA-4 inhibitor. A phase 2 trial enrolled 30 patients with mCRPC [47]. With a median follow-up of 45.5 months, median radiographic PFS and OS were 3 months and 24.3 months, respectively. Overall, 28% of patients receiving treatment experienced grade 3 adverse effects (AE) without any grade 4–5 cases. A favorable cohort has been identified expressing a higher density of cytotoxic and memory T cells in the tumor and an increased expression of IFN-γ signaling suggesting the potential role of these biological markers as theranostic factors.

Ipilimumab has also been evaluated in a phase 3 trial (CA184-095) randomizing 602 chemonaïve patients with asymptomatic or minimally symptomatic mCRPC without visceral metastasis to receive ipilimumab or placebo in a 2:1 ratio [48]. No significant difference in OS was observed between arms (27.8 vs. 29.7 months, respectively). However, ipilimumab was associated with a longer median PFS (5.6 vs 3.8 months). AEs grade 4–5 were observed in 27% patients in ipilimumab group versus 2% in the placebo arm. This large trial did not conclusively demonstrate an ipilimumab-driven benefit for OS.

Loss of function in mismatch repair (MMR) genes has been associated with favorable responses to PD-1 blockade immunotherapy in different cancer types including PCa [49]. In a case series, anti PD1/PD-L1 was used in 11 men with mCRPC. Overall, five patients had durable clinical benefit, five had no benefit, and one had stable disease for approximately 6 months [50].

Pembrolizumab has been assessed in the phase 2 KEYNOTE-199 study among 258 patients with mCRPC previously treated with docetaxel and at least one hormonal therapy [51]. Cohort 1 and 2 included any measurable disease with PD-L1 positive and PD-L1 negative patients, cohort 3 included patients with bone predominant disease, regardless of PD-L1 expression. Median follow-up was 16.8 months. Objective response rate (ORR) was 5% in cohort 1 and 3% in cohort 2 with a median OS of 9.5 and 7.9 months, respectively. Median OS in cohort 3 was 14.1 months. Pembrolizumab monotherapy showed encouraging results for antitumor activity and disease control with an acceptable safety profile, pushing for additional investigations. Based on these results, Food and Drug Administration (FDA) approved pembrolizumab for the treatment of mCRPC with MMR deficiency or high microsatellite instability.

Avelumab is a PD-L1 inhibitor. The phase 1 trial reported in mCRPC patients included 18 patients, while seven had stable disease after 24 weeks of treatment [52]. Avelumab was safe and tolerable with 15 patients experiencing grade 1–2 AEs and only one grade 3 AE. Immune analysis and other studies are awaited to determine which patients would benefit most from this treatment.

### Combinations

#### Immunotherapy combination

Nivolumab, a PD-1 inhibitor, has been combined with ipilimumab in different cancers. CheckMate 650 is a phase 2 study of nivolumab plus ipilimumab for the treatment of mCRPC [53]. This study involved two cohorts. The first cohort included asymptomatic or minimally symptomatic patients who progressed after second generation hormone therapy (no prior chemotherapy) and the second cohort included patients who progressed after taxane-based therapy. Objective response rate (ORR) was 26% in cohort 1 and 10% in cohort 2. PSA decline > 50% was observed in 21% of cases in cohort 1 and 13% of cases in cohort 2. A PD-L1 ≥ 1% expression, the presence of DNA damage repair, a homologous recombination deficiency, or an above-median tumor mutational burden were associated with higher ORR. Grade 3–4 AEs occurred in 39 and 51% of patients in cohorts 1 and 2, respectively, with one death reported in each cohort.

Another phase 2 trial used nivolumab plus ipilimumab for patients with ARV7^+^ mCRPC [54]. Overall, 15 patients were enrolled. With a median follow-up of 8.4 months, 7% had PSA response rate and ORR was 25%. OS was 9.5 months, whereas patients with DNA repair deficient tumors had a more favorable biochemical and radiographic PFS. This combination revealed acceptable safety and encouraging efficacy, particularly in men with DNA repair deficient tumors.

CTLA-4 blockade could enhance antitumor immunity when combined with cancer vaccines. A phase 1–2 trial included 28 patients with asymptomatic chemonaive mCRPC treated with a combination of GVAX and ipilimumab [55]. OS was 29 months and 32.1% patients had a PSA response.

Pre-treatment high levels of CD4^+^_,_ CTLA-4^+^, CD4^+^PD-1^+^, non-naive CD8^+^ and low frequencies of CD4^+^ or regulatory T cells were associated with a significantly prolonged OS. OS was extended when treatment induced > 25% increase in lymphocyte counts, > 30% increase in non-naive (memory) CD4^+^ T cells, CD4^+^ and CD8^+^ T-cell activation.

### ART and immunotherapy

ART such as enzalutamide might improve IFN-γ levels and sensitize tumor cells to immune-mediated cell-killing. Immunotherapy associated with enzalutamide seems to be an interesting combination for patients with PCa.

Atezolizumab is a humanized monoclonal antibody which inhibits the interaction between PD-L1 and its receptor. The combination of atezolizumab and enzalutamide has been examined in the phase III randomized trial IMbassador250 evaluating the combined treatment vs enzalutamide alone in 759 mCRPC or locally advanced CRPC patients who had progressed on abiraterone and docetaxel, or who were not candidates for a taxane regimen. Overall survival was not different between arms discouraging the use of the combination [56].

In the above-mentioned multicohort phase 2 study KEYNOTE-199, the cohorts 4 (measurable disease) and 5 (bone predominant disease) received a combination of pembrolizumab and enzalutamide [57]. After a median follow-up of 13.7 months, most patients had disease progression. Disease control rate was 51%. ORR for patients with measurable disease was with 12% relatively low. However, duration of response was almost 6 months in 60% of responders.

### Immunotherapy and PARP inhibitor

Durvalumab is a human IgG1-K monoclonal antibody that targets PD-L1. Olaparib is a PARP inhibitor approved for patients with mCRPC carrying homologous recombination repair gene alteration. Preclinical data have suggested a synergistic effect between PARP and checkpoint inhibitors. A phase II, open-label trial has assessed this combination in multiple cohorts of heavily pretreated mCRPC patients [58]. Seventeen patients were enrolled and received durvalumab plus olaparib. Median rPFS was 16.1 months. The 1-year PFS rate was 83.3% for patients with alteration in DNA damage response gene vs. 36.4% for those without mutations (*p* = 0.031). Most common treatment-related grade 3 or 4 AEs were anemia, lymphopenia, infection, and nausea.

### Immunotherapy and local treatments

Radiation treatment may have a systemic role by activating the immune system, stimulating immune priming (abscopal effect), and improving response to immunotherapy.

A phase 1–2 study assessing ipilimumab ± radiotherapy in mCRPC disease showed a 29% rate of non-progressive disease with the treatment combination after a median follow-up of 15.7 months [59]. In this trial, metastasis-directed radiotherapy was given at a single dose of 8 Gy per target bone lesion (for up to three lesions per patient) at 24–48 h before the first ipilimumab dose.

A phase 3 study included patients with post-docetaxel mCRPC and bone metastasis and randomized patients to receive bone-directed radiotherapy with ipilimumab or placebo [60]. All patients received a single dose of radiotherapy of 8 Gy for at least one, and up to five, bone lesions. Radiotherapy was done within the 2 days before initiation of the study drug regimen, and palliative radiotherapy was allowed for any bone lesion while on study. The median follow-up was 9.9 months in ipilimumab group and 9.3 months in placebo group. Median overall survival was 11.2 and 10.0 months [HR 0.85], respectively, without significant difference (*p* = 0.053). Median PFS was 4.0 months for ipilimumab group vs. 3.1 for placebo group [HR 0.70, *p* < 0.001]. Post hoc analysis suggested that ipilimumab might provide the most benefit in patients with favorable prognostic features, specifically in patients without visceral metastasis. The rates of grade 3–4 AEs were 59% for ipilimumab group vs 41% for placebo group.

Cryotherapy might also help to increase the immunogenicity of PCa. A pilot trial has evaluated the role of prostate cryotherapy, short term androgen deprivation and pembrolizumab in oligometastatic PCa [61]. After 1 year, 42% of patients had a PSA level below 0.6 ng/mL for a median PSA-PFS of 14 months. Median systemic therapy-free survival was 17.5 months. No grade 3 or more AEs was observed.

### Ongoing trials

Multiple clinical trials are ongoing assessing immunotherapy drugs and vaccines for the management of prostate cancer (Table 2). IT is currently evaluated alone or in combination with life-prolonging or investigational drugs, from localized to late-stage mCRPC.

**Table 2:** Main phase 2 and 3 ongoing immunotherapy trials (NCT.gov)

Clinical trial	Drug	Phase	Estimated enrolment	Population	Primary endpoint	Arms	Estimated completion date
NCT03686683	Sipuleucel-T	3	450	Low risk localized prostate cancer	Proportion of subjects without histological upgrading within 36 months	Arm 1: Sipuleucel-T Arm 2: active surveillance	May 2023
NCT02649439	PROSTVAC	2	98	Biochemically recurrent PCa	Time to progression	Arm 1: PROSTVAC at recurrence Arm 2: PROSTVAC 6 months after recurrence	October 2021
NCT03579654	Proscavax	2	120	Low risk localized prostate cancer	PSA DRE	Arm 1: Proscavax treatment Arm 2: active surveillance	June 2022
NCT03506997 PERSEUS1	Pembrolizumab	2	100	mCRPC with high mutational load	ORR CTC count PSA50		September 2025
NCT03248570	Pembrolizumab	2	50	mCRPC	rPFS	Arm 1: DDR proeficient DDR deficient	March 2023
NCT04104893 CHOMP	Pembrolizumab	2	30	mCRPC with dMMR or CDK12-/-	PSA50 ORR		March 2023
NCT03179410 PICK-NEPC	Avelumab	2	15	Metastatic neuroendocrine-like PCa	ORR		January 2023
Combination							
Combination with vaccines							
NCT01804465	Sipuleucel-T Ipilimumab	2	50	mCRPC	Immune response toxicity	Arm 1: Ipilimumab started 1 day after sipuleucel-T Arm 2: Ipilimumab started 3 weeks after sipuleucel-T	October 2021
NCT01818986	Sipuleucel-T SABR	2	20	mCRPC	Time to progression		December 2024
NCT03315871	PROSTVAC, CV301, and MSB0011359C	2	34	Biochemically recurrent PCa	30% decline in PSA at 6 and 12 months		December, 2023
NCT01867333	PROSTVAC Enzalutamide	2	57	mHSPC	Time to progression		January 2022
NCT02768363 ULYSSES	ProstAtak®(AdV-tk) Valacyclovir	2	187	Patients undergoing active surveillance for localized prostate cancer	Proactive surveillance score at 12 months	Arm 1: ProstAtak® (AdV-tk) + valacyclovir Arm 2: Placebo + valacyclovir +	September 2020
NCT01436968 PrTK03	ProstAtak®(AdV-tk) Valacyclovir	3	711	Intermediate-high risk localized prostate cancer (standard prostate-only radiation therapy)	Disease FS	Arm 1: ProstAtak® (AdV-tk) + valacyclovir + radiation therapy ± ADT Arm 2: Placebo + valacyclovir + radiation therapy ± ADT	December 2022
Immunotherapy combination							
NCT03570619 IMPACT	Nivolumab + Ipilimumab	2	40	CDK12 loss of function metastatic CRPC	ORR		September 2021
NCT02649855	Nivolumab Ipilimumab	2	175	mCRPC with immunogenic signature	ORR		July 2025
NCT02788773	Durvalumab Tremelimumab	2	52	mCRPC	ORR	Arm 1: durvalumab + tremelimumab Arm 2: durvalumab alone	December 2020
NCT04336943	Durvalumab and olaparib	2	30	Biochemically recurrent PCa predicted to have a high neoantigen load	Undetectable PSA		April 2024
NCT04159896	ESK981 and nivolumab	2	49	mCRPC	PSA50 Safety and tolerability		March 2022
Immunotherapy and chemotherapy or second generation hormonotherapy							
NCT03879122 PROSTRATEGY	Ipilimumab Nivolumab Docetaxel ADT	2/3	135	mHSPC		Arm 1: ADT plus 6 cycles of DOCETAXEL Arm 2: ADT plus DOCETAXEL plus NIVOLUMAB Arm 3: ADT plus IPILIMUMAB alternating with DOCETAXEL and with NIVOLUMAB	December, 2023
NCT03338790 CheckMate 9KD	Nivolumab Docetaxel Enzalutamide Rucaparib	2	330	mCRPC	ORR RR-PSA	Arm 1: nivolumab + rucaparib Arm 2: nivolumab + docatexel + prednisone Arm 3: nivolumab + enzalutamide	November 2021
NCT03834506 KEYNOTE 921	Pembrolizumab Docetaxel	3	1000	mCRPC	OS rPFS	Arm 1: pembrolizumab + docetaxel Arm 2: Placebo + docetaxel	February 2023
NCT04191096 KEYNOTE 991	Pembrolizumab Enzalutamide ADT	3	1232	mHSPC	rPFS OS	Arm 1: pembrolizumab + enzalutamide + ADT Arm 2: placebo + enzalutamide + ADT	September 2026
NCT03834493 KEYNOTE 641	Pembrolizumab Enzalutamide	3	1200	mCRPC	OS rPFS	Arm 1: Pembrolizumab + enzalutamide Arm 2: Placebo + enzalutamide	April 2024
NCT03834519 KEYLYNK-010	Pembrolizumab Olaparib Abiraterone acetate Enzalutamide	3	780	mCRPC	OS rPFS	Arm 1: pembrolizumab + olaparib Arm 2: Abiraterone acetatone or enzalutamide	September 2022
NCT04262154	Atezolizumab Abiraterone acetate Lupron Radiation therapy	2	44	mCRPC	Failure-free		September 2022
NCT01688492	Ipilimumab + abiraterone acetate	2	57	mCRPC	PFS Safety	Ipilimumab + abiraterone acetate	September 2020
NCT04446117 CONTACT-02	Cabozantinib Atezolizumab Abiraterone acetate Enzalutamide	3	580	mCRPC	OS PFS	Arm 1: cabozantinib + tezolizumab Arm 2: Abiraterone or enzalutamide	July 2023
Immunotherapy and radiotherapy							
NCT03543189	Nivolumab + Brachytherapy + External Beam Radiation	1/2	34	Oligometastatic HSPC	Safety RFS		September 2021
NCT03795207 POSTCARD	Stereotactic Body Radiation Therapy Durvalumab	2	96	Oligometastatic relapse following treatment with curative intent	PFS	Arm 1: Stereotactic Body Radiation Therapy + Durvalumab Arm 2: Stereotactic Body Radiation Therapy	September 2024
Immunotherapy and radical prostatectomy							
NCT03753243	Neoadjuvant Pembrolizumab + Enzalutamide before radical prostatectomy	2	32	High risk localized PCa	Pathologic complete response		September 2025
NCT02020070	Ipilimumab Degarelix Radical prostatectomy	2	16	Oligometastatic HSPC	Undetectable PSA	Arm 1: Ipilimumab + degarelix and radical prostatectomy Arm 2: Ipilimumab + degarelix prior to radical prostatectomy	December 2021

*ADT* androgen deprivation therapy; *DDR* DNA damage repair; *DRE* digital rectal examination; *HDR* high dose rate; *mHSPC* metastatic hormone-sensitive prostate cancer; *OS* overall survival; *ORR* overall response rate; *PCa* prostate cancer; *PFS* progression free survival; *PSA* prostate specific antigen; *PSA50*  > 50% PSA response rate; *RR* response rate; *RFS* relapse free survival; *rPFS* radiologic progression free survival

However, to date, even if many strategies are promising, sipuleucel-T and pembrolizumab remain the only IT strategies approved by the FDA without widespread use in routine practice. Management of metastatic PCa is a rapidly evolving field. Recently, several drugs have been proven to be life-prolonging, including new hormone therapies, theranostic radioligands and PARP inhibitors. The choice of the ideal treatment for an individual patient will be probably guided in a close future by the validation of predictive factors of response/resistance to avoid ineffective therapy and to prolong tumor response. By considering that perspective, the use of immunotherapy could be driven by the quantification of lymphocytes populations with the example of the immunoscore developed in colorectal cancer [6–8]. The level of expression of immunoregulators in tumor tissue could be also relevant to anticipate response to immune checkpoints inhibitors targeting PD-1/PD-L1 or CTLA4. However, to date, no biological or immunological signature has been validated in PCa to guide this immunotherapy choice.

## Conclusions

The relevance of IT strategies in the treatment course of PCa is still ambiguous. Progress in translational research and results from ongoing large 2 and 3 trials are urgently awaited to draw clinically applicable conclusions.
