# Correction to: Immunotherapy in prostate cancer: new horizon of hurdles and hopes

**DOI:** 10.1007/s00345-021-03849-5

**Published:** 2021-10-13

**Authors:** Igor Tsaur, Maximilian P. Brandt, Eva Juengel, Cécile Manceau, Guillaume Ploussard

**Affiliations:** 1 Department of Urology and Pediatric Urology, University Medical Center, Johannes Gutenberg University, Langenbeckstr. 1, 55131 Mainz, Germany; 2 Department of Urology, CHU-Institut Universitaire du Cancer Toulouse—Oncopole, Toulouse, France; 3 Department of Urology, La Croix du Sud Hospital, Toulouse, France

## Correction to: World Journal of Urology (2021) 39:1387–1403 10.1007/s00345-020-03497-1

The article “Immunotherapy in prostate cancer: new horizon of hurdles and hopes” written Tsaur, I., Brandt, M.P., Juengel, E., Manceau, C., Ploussard, G. by was originally published Online First without Open Access. After publication in volume 39, issue 5, pages 1387–1403 the author decided to opt for Open Choice and to make the article an Open Access publication. Therefore, the copyright of the article has been changed to © The Author(s) 2020 and the article is forthwith distributed under the terms of the Creative Commons Attribution 4.0 International License, which permits use, sharing, adaptation, distribution and reproduction in any medium or format, as long as you give appropriate credit to the original author(s) and the source, provide a link to the Creative Commons licence, and indicate if changes were made. The images or other third party material in this article are included in the article’s Creative Commons licence, unless indicated otherwise in a credit line to the material. If material is not included in the article’s Creative Commons licence and your intended use is not permitted by statutory regulation or exceeds the permitted use, you will need to obtain permission directly from the copyright holder. To view a copy of this licence, visit http://creativecommons.org/licenses/by/4.0.

The original article has been corrected.

